# A Healthful Plant-Based Diet Is Associated with Lower Odds of Nonalcoholic Fatty Liver Disease

**DOI:** 10.3390/nu14194099

**Published:** 2022-10-02

**Authors:** Xiude Li, Zhaohong Peng, Meiling Li, Xueke Zeng, Haowei Li, Yu Zhu, Hui Chen, Anla Hu, Qihong Zhao, Zhuang Zhang, Hua Wang, Changzheng Yuan, Wanshui Yang

**Affiliations:** 1 Department of Nutrition, School of Public Health, Anhui Medical University, Hefei 230032, China; 2 Key Laboratory of Population Health Across Life Cycle, Anhui Medical University, Ministry of Education of the People’s Republic of China, Hefei 230032, China; 3 NHC Key Laboratory of Study on Abnormal Gametes and Reproductive Tract, Hefei 230032, China; 4 Anhui Provincial Key Laboratory of Population Health and Aristogenics/Key Laboratory of Environmental Toxicology of Anhui Higher Education Institutes, Anhui Medical University, Hefei 230032, China; 5 Department of Interventional Radiology, the First Affiliated Hospital of Anhui Medical University, Hefei 230032, China; 6 Department of Big Data and Health Science, School of Public Health, Zhejiang University School of Medicine, Hangzhou 310058, China; 7 Department of Oncology, the First Affiliated Hospital of Anhui Medical University, Anhui Medical University, Hefei 230032, China

**Keywords:** plant-based diet, controlled attenuation parameter, nonalcoholic fatty liver diseases, body mass index

## Abstract

There is little evidence for the associations of the overall plant-based diet index (PDI), the healthful PDI (hPDI), and the unhealthful PDI (uPDI) with the odds of nonalcoholic fatty liver disease (NAFLD). We present a nationwide cross-sectional study among US adults aged 18 years or older. Diet was assessed by 24-h recalls. Overall PDI, hPDI, and uPDI were constructed based on 18 food groups. NAFLD was defined based on controlled attenuation parameter derived via transient elastography (TE) in the absence of other causes of chronic liver disease. Among 3900 participants with eligible TE examination, 1686 were diagnosed with NAFLD. The overall PDI was not associated with NAFLD prevalence (comparing extreme tertiles of PDI score OR = 1.03, 95% CI 0.76, 1.38, *p*_trend_ = 0.609). However, hPDI was inversely (OR = 0.50, 95% CI 0.35, 0.72, *p*_trend_ < 0.001), while uPDI was positively associated with odds of NAFLD (OR = 1.37, 95% CI 0.93, 2.02, *p*_trend_ = 0.009) in the multivariable-adjusted models without body mass index (BMI). After further adjustment for BMI, only the association of hPDI with NAFLD remained statistically significant (OR = 0.64, 95% CI 0.46, 0.87, *p*_trend_ = 0.006). Such inverse association appeared stronger in non-Hispanic whites, but not in other racial/ethnic groups (*p*_interaction_ = 0.009). Our findings suggest that a plant-based diet rich in healthy plant foods might be associated with lower odds of NAFLD, particularly among US non-Hispanic whites. Clinical trials and cohort studies to validate our findings are needed.

## 1. Introduction

Nonalcoholic fatty liver disease (NAFLD) has become the major cause of chronic liver diseases and affects approximately 30% of the US population [1]. This threat is aggravated by the fact that no drugs have been approved to treat such diseases. Therefore, lifestyle interventions including dietary modifications are key to manage NAFLD [2]. For example, the Mediterranean diet, characterized by high consumption of a plant-based diet is highly recommended for preventing and managing NAFLD [3].

Nonetheless, several but not all observational studies [4,5,6,7] have suggested a beneficial effect of plant-based diets on NAFLD. These studies are somewhat limited because most [4,5,6] defined plant-based diets as vegetarian diets, which excluded some or all of the animal foods, and did not distinguish healthy plant foods from less healthy plant foods. To completely give up some or all animal foods to become vegetarian is difficult for many. Therefore, investigating the effect on health of progressively increasing plant foods while reducing animal foods is important from the public health perspective. In addition, not all plant foods have health benefits [8,9,10]. For example, increased consumption of some less healthy plant foods, such as starchy vegetables and fruit juice, may be positively associated with chronic liver disease or mortality [11,12]. To address these limitations, Satija et al. [8] developed three dietary indices, including an overall plant-based diet index (PDI), a healthful PDI (hPDI), and an unhealthful PDI (uPDI). Overall PDI can represent a gradually increasing intake of plant foods and concomitantly reducing consumption of animal foods. hPDI, which emphasizes intake of healthy plant foods, and uPDI, which emphasizes intake of less healthy plant foods, can address the weakness when all of the plant foods are treated as the same.

However, few studies to date have assessed the associations of PDIs with NAFLD or fatty liver disease (FLD) [7,13,14,15]. For example, one study with a cross-sectional design investigated PDIs in relation to NAFLD among US adults, in which NAFLD was determined based on fatty liver index (FLI) derived from combining waist circumference, triglyceride, gamma-glutamyl-transferase, and body mass index (BMI) [7]. Another cross-sectional study assessed the associations of PDIs with magnetic resonance imaging (MRI)-assessed FLD in a small population (*n* = 578) in northern Germany [13]. To our knowledge, however, the associations of PDIs with the transient elastography (TE)-assessed NAFLD, with TE being one of the most valid methods to detect and grade hepatic steatosis [16,17], have not yet been evaluated. In addition, it is not yet clear whether a non-linear relationship between PDIs and NAFLD exists, although prior studies have observed non-linear associations between several foods or dietary patterns and multiple health outcomes [10,18].

Therefore, we hypothesized that overall PDI, especially hPDI, might be inversely associated with NAFLD, while uPDI might be positively associated. To test this hypothesis, we investigated PDIs (i.e., the overall PDI, hPDI, uPDI) in relation to the odds of TE-assessed NAFLD among US adults, using a large nationally representative cross-sectional data from the 2017–2018 cycle of the National Health and Nutrition Examination Survey (NHANES) in which TE examination was conducted for the first time in a nationwide survey. We also evaluated the potential non-linear association of PDIs with the odds of NAFLD.

## 2. Methods

### 2.1. Study Population

NHANES is a continuous, cross-sectional nationwide survey designed to assess the health and nutritional status of a sample representative of the civilian noninstitutionalized household population of all ages in the US. Approximately 5000 persons were enrolled annually in the survey. Details on NHANES design have been reported elsewhere [19]. All participants provided written informed consent. Study protocols were approved by the National Centers for Health Statistics (NCHS) Research Ethics Review Board (Protocol #2011-17; Protocol #2018-01). 

Data were analyzed in the 2017–2018 cycle of NHANES (*n* = 9254). Participants were excluded if they (i) were younger than 18 years (*n* = 3398); (ii) did not have 24-h dietary interview (*n* = 873); (iii) had implausible values for energy intake (i.e., <600 or >3500 kcal/day for women and <800 or >4200 kcal/day for men, *n* = 221); (iv) did not have TE data (*n* = 70); (v) had unreliable TE examination results (*n* = 522); (vi) were suffering from hepatitis B and/or C (*n* = 91); (vii) reported significant alcohol consumption (i.e., >2 drinks/day for women and >3 drinks/day for men, *n* = 105), or (viii) were taking steatogenic medications (i.e., amiodarone, valproate, methotrexate, tamoxifen, and corticosteroid) for at least 3 months or more before enrollment (*n* = 74) [2,20]. After exclusion, 3900 participants (2028 women and 1872 men) were finally included in the analyses (Figure S1).

### 2.2. Dietary Assessment and Diet Indices

Diet was assessed by 24-h dietary recalls. The multiple-pass method was used to improve complete and accurate data collection and decrease the respondent burden [21]. In the current analysis, all participants had completed the first recall, which was conducted in-person in the NHANES Mobile Examination Center (MEC). Most participants (*n* = 3434, 88.1%) had completed a second recall, which was performed by telephone 3 to 10 days after the first recall. 

Details on the construction of the three PDIs have been described previously [8,9,10]. In short, 18 food groups (Table S1) were created based on their similarities in nutrients and culinary use. We then aggregated these food groups into three broad categories: healthy plant foods, less healthy plant foods, and animal foods. Notably, fruit juices are rich in natural sugars and may have a negative health effect similar to sugar-sweetened beverages (SSBs) [22], and thus were classified into less healthy plant-based foods. Because alcohol drinking has different effects on health, and the fatty acid composition of margarine changes over time, these foods were not included. The intake of all the food groups was ranked into quintiles [8,9,10]. For overall PDI, both the healthy and less healthy plant foods received positive scores (i.e., scores from one (the lowest quintile) to five (the highest quintile)), whereas the animal foods received reverse scores (i.e., scores from five (the lowest quintile) to one (the highest quintile)). For hPDI, the healthy plant foods received positive scores, whereas both the less healthy plant foods and animal food received reverse scores. Conversely, the less healthy plant foods received positive scores, whereas both the healthy plant foods and animal foods were given the reverse scores for uPDI. Finally, scores of the PDIs were the sum of the scores of 18 food groups and ranged from 18 to 90. A higher overall PDI score indicated a greater intake of all types of plant foods. A higher hPDI score represented increased consumption of healthy plant foods and less consumption of less healthy plant foods. Conversely, a higher uPDI score implies lower healthy plant food intake and greater less healthy plant food consumption.

The reproducibility and validity of the three PDIs have been reported elsewhere [23,24]. Briefly, comparing the three PDIs derived from questionnaire with those from the average of two 7-d dietary records, the Spearman correlation coefficients ranged from 0.63 to 0.78 in a US population [23]. In addition, the PDIs derived from 24-h dietary recalls showed acceptable face validity and construct validity [24].

### 2.3. Ascertainments of NAFLD

In the 2017–2018 NHANES cycle, vibration-controlled TE was conducted by trained technicians using a FibroScan^®^ 502 V2 Touch model equipped with a medium or extra-large wand (probe). Consistent with the previous studies [16,25], NAFLD was assessed by the TE-derived controlled attenuation parameter (CAP) with a CAP cut-off value of 274 dB/m (≥S1). By comparing CAP measurement for the detection of steatosis against biopsy, the area under receiver operating characteristic (AUROC) curves was 0.87 (95% confidence interval (CI) 0.82, 0.92) with a sensitivity and specificity of both 90% for S ≥ S1 among NAFLD patients [16].

### 2.4. Assessments of Covariates

A household interview was conducted to collect information on demographic factors (e.g., sex, educational level, age, race/ethnicity, and income) and lifestyle factors (e.g., physical activity and smoking). Data on weight, height, and alcohol intake were collected from persons who received physical examinations in the MEC. We used the ratio of family income to poverty, defined as the family income divided by poverty thresholds, to assess income level. Physical activity was estimated in metabolic equivalent tasks (METS) hours per week. Hepatitis B virus infection was defined if individuals were hepatitis B surface antigen (HBsAg)-positive, while hepatitis C virus infection was determined by both hepatitis C antibody and RNA positivity. Diabetes was defined by a self-report of diagnosis of diabetes, a fasting glucose level of ≥126 mg/dL, or a hemoglobin A1c (HbA1c) level of ≥6.5%. Prediabetes was defined as self-reported prediabetes, or fasting glucose of 100–125 mg/dL, or HbA1c of 5.7–6.4%. Laboratory methods of assessing HBsAg, hepatitis C antibody and RNA, fasting glucose, and HbA1c are described elsewhere [26].

### 2.5. Statistical Analysis

To ensure nationally representative estimates, all analyses in the current study incorporated sampling weights, stratification, and clustering of the complex sampling design. The prevalence of NAFLD was standardized by the 2020 US population. Multiple logistic regression was used to calculate the odds ratios (ORs) and 95% CIs for NAFLD associated with the three PDIs. Results are presented for the following three models. Model 1 was adjusted for age only. Model 2 was further adjusted for sex, ratio of family income to poverty, race/ethnicity, total energy intake, marital status, education, smoking, alcohol drinking, diabetes, and physical activity. Given that BMI is a potential mediator in the association of PDIs with the odds of NAFLD, we additionally adjusted for BMI in a separate model (i.e., model 3). We selected the above covariates (see categorizations in Table S2) based on professional knowledge, previously identified risk factors for NAFLD, and the observed incomparability of participant characteristics. We put covariates including total energy intake, age, BMI, ratio of family income to poverty, and physical activity, into the models as categorical variables, considering that these variables may have a non-linear association with odds of NAFLD. A missing value indicator was created for each covariate in the models, if possible. We presented ORs by tertile categories and per 10–point increase in each PDI, and a linear trend test was performed by treating each PDI as a continuous variable in the models. Restricted cubic splines were used to investigate the possible non-linear relationships of the PDIs with the prevalence of NAFLD.

To examine the robustness of the results, we repeated the analysis using a CAP cut-off value of ≥ 288 dB/m [27] or using the US FLI (≥30) [28] to define NAFLD. We also repeated the analysis in people not having diabetes or prediabetes, or by treating the numerical covariates as continuous variables. In addition, we calculated the NAFLD fibrosis score (NFS) and fibrosis-4 index (FIB-4) to identify liver fibrosis in patients with NAFLD, and then assessed the associations of PDIs with high NFS (>0.676) [29] and high FIB-4 (>3.25) [30]. In addition, given that combinations of BMI and waist circumference may better account for obesity than BMI alone, we adjusted for waist circumference. In a subgroup analysis, we stratified the associations between PDIs and odds of NALFD by potential confounders. The Wald test was used to assess the interaction terms between PDIs and these potential confounders. We used Bonferroni correction to determine the threshold of significance with *p* < 0.017 (0.05/3 exposures × 1 outcome) for main analysis and *p* < 0.005 (0.05/(1 exposure × 1 outcome × 11 groups)) for subgroup analysis to account for multiple comparisons. The statistical power was over 90% for both hPDI and uPDI with a type I error probability of 0.05 in the current analysis, which was calculated using the method suggested by Dupont and Plummer [31]. All statistical analyses were two-sided and conducted with SAS version 9.4 (SAS Institute Inc., Cary, NC, USA).

## 3. Results

### 3.1. Participants’ Characteristics

The mean age of participants in this study was 49.2 years (SD 18.4, ranged 18–80). A total of 1686 (age-standardized prevalence = 42.5%) were diagnosed with NAFLD. Participants with higher overall PDI scores generally consumed more plant foods, but SSBs and less animal foods except for fish and seafood. Participants with higher hPDI scores consumed more healthy plant foods and lower animal and less healthy plant foods, with the exception of fish and seafood. Conversely, participants with higher uPDI scores had a higher intake of less healthy plant foods but consumed less animal and healthy plant foods except for miscellaneous animal-based foods (Table S3). Participants with higher overall PDI or hPDI scores had lower BMI, were older, were more likely to be female and married, had a higher level of education and income level, and were less likely to be current smokers, whereas reversed trends were observed for uPDI (Table 1).

### 3.2. PDIs and NAFLD

**Figure 1:**
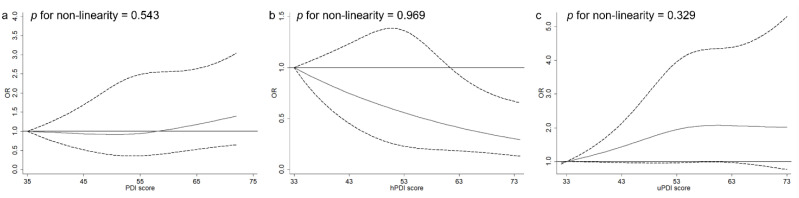
Spline analysis for the association of overall PDI (**a**), hPDI (**b**), uPDI (**c**), with the odds of NAFLD^a^. hPDI, healthful plant-based diet index; NAFLD, nonalcoholic fatty liver disease; PDI, plant-based diets index; uPDI, unhealthful plant-based diet index. ^a^ The models were adjusted for the same covariates listed for model 3 in Table 2.

In multivariable-adjusted models without BMI (Table 2), a higher hPDI score was associated with lower odds of NAFLD (comparing extreme tertiles OR = 0.50, 95% CI 0.35, 0.72, *p*_trend_ < 0.001), while uPDI was associated with higher odds of NAFLD (OR = 1.37, 95% CI 0.93, 2.02, *p*_trend_ = 0.009). Upon further adjustment for BMI, only the association of hPDI with NAFLD remained statistically significant (OR = 0.64, 95% CI 0.46, 0.87, *p*_trend_ = 0.006). The association between uPDI and NAFLD was largely attenuated and lost statistical significance after additionally controlling for BMI (OR = 1.14, 95% CI 0.79, 1.66, *p*_trend_ = 0.173). A non-significant association between overall PDI and NAFLD was observed (OR = 1.03, 95% CI 0.76, 1.38, *p*_trend_ = 0.609). In the restricted cubic spline model, non-linear relationships between PDIs and NAFLD were not observed (*p* for non-linearity > 0.05, Figure 1).

### 3.3. Sensitivity and Subgroup Analysis

The results of the sensitivity analyses were consistent with the main analysis when using different CAP cut-off value to define NAFLD (Table S4), with ORs (per 10-point increase in dietary pattern score) of 0.72 (95% CI 0.55, 0.94, *p*_trend_ = 0.014) for hPDI and 1.11 (95% CI 0.86, 1.42, *p*_trend_ = 0.429) for uPDI, or using US FLI to define NAFLD (Table S5), with ORs of 0.64 (95% CI 0.44, 0.92, *p*_trend_ = 0.017) for hPDI and 1.41 (95% CI 1.03, 1.91, *p*_trend_ = 0.026) for uPDI, or restricting analysis in people not having diabetes or prediabetes (Table S6), with ORs of 0.72 (95% CI 0.57, 0.91, *p*_trend_ = 0.007) for hPDI and 1.26 (95% CI 0.97, 1.64, *p*_trend_ = 0.080) for uPDI, or repeating analysis by treating the numerical covariates as continuous variables (Table S7), with ORs of 0.70 (95% CI 0.52, 0.95, *p*_trend_ = 0.020) for hPDI and 1.28 (95% CI 1.03, 1.60, *p*_trend_ = 0.027) for uPDI, or additionally adjusting for waist circumference (Table S8), with ORs of 0.76 (95% CI 0.60, 0.97, *p*_trend_ = 0.028) for hPDI and 1.12 (95% CI 0.88, 1.41, *p*_trend_ = 0.350) for uPDI. In addition, we found that hPDI was inversely associated (OR = 0.64, 95% CI 0.43, 0.96, *p*_trend_ = 0.030), whereas uPDI was positively (OR = 1.86, 95% CI 1.20, 2.88, *p*_trend_ = 0.005) associated with high NFS (Table S9). We only observed an inverse association between the overall PDI and high FIB-4 (Table S10).

**Figure 2:**
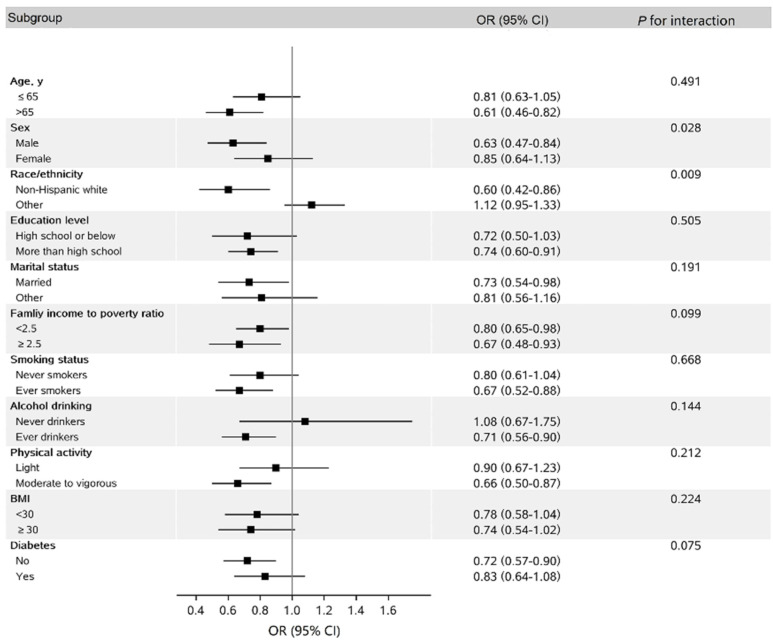
Subgroup analysis for the association between hPDI scores (per 10–point increase) and odds of NAFLD ^a^. BMI, body mass index; CI, confidence interval; hPDI, healthful plant-based diet index; METS, metabolic equivalent tasks; NAFLD, nonalcoholic fatty liver disease; OR, odds ratio. ^a^ The models were adjusted for the same covariates as those listed for model 3 in Table 2 except for the variables examined in this figure. Physical activity <8.3 METS–h/week was defined as light physical activity, and ≥8.3 METS–h/week was defined as moderate to vigorous activity. Participants with any missing values in covariates were excluded from the subgroup analysis.

In stratified analysis, we found that the inverse association between hPDI and NAFLD seemed stronger in non-Hispanic whites than in others, with borderline significance (*p*_interaction_ = 0.009, Figure 2).

## 4. Discussion

In this study, we found that following hPDI might be associated with lower prevalence, while uPDI might be associated with a higher prevalence, of TE-assessed NAFLD. These associations were all diluted after adjusting for BMI, and only the association of hPDI with NAFLD remained statistically significant. In addition, the inverse association for hPDI might be dependent on race/ethnicity, with a stronger association being observed in non-Hispanic whites than in other ethnic groups.

Observational studies of PDIs with NAFLD or FLD are limited and have yielded inconsistent results [7,13,14,15]. Notably, a cross-section study [7] also used NHANES data (i.e., NHANES 2005–2010), and found significant inverse associations of both overall PDI (the highest vs. lowest third of PDIs scores OR = 0.79) and hPDI (OR = 0.76) with odds of NAFLD, and showed a positive association for uPDI (OR = 1.34), which were partly in line with our results. These significant associations remained even after adjusting for BMI. However, this study [7] determined NAFLD based on FLI. Different from this approach, our study was able to derive CAP through TE to define NAFLD with higher sensitivity and specificity [14,32]. Similar results were obtained in a recent study of 3042 subjects, assessing NAFLD through hepatic steatosis index (HSI), an algorithm combining alanine transaminase, aspartate transaminase, BMI, sex, and type 2 diabetes [14]. Nevertheless, another cross-sectional study [13] in northern Germany used liver signal intensity (LSI) via MRI to define FLD, and did not find any significant associations between PDIs and FLD. Although the MRI method is more accurate than CAP in detecting and grading steatosis in NAFLD patients [33], a small sample size (*n* = 578) with limited FLD cases (*n* = 231) in the latter study [13] may have hampered the conclusions. In addition, consistent with our findings, a recent cross-section study used non-contrast CT scans to define fatty liver and only found an inverse association between hPDI and fatty liver (OR per 5-score increment = 0.76) [15]. The above inconsistency could be partly due to differences in the outcome (i.e., NAFLD vs. FLD), study population, sample size, and methods for outcome ascertainment. Due to the cross-sectional design, clinical trials or cohort studies are warranted to confirm our findings.

Findings in the present study are somewhat consistent with previous studies that reported an inverse association between intake of vegetables and fruits, and other healthful plant foods such as whole grains and nuts (the main source of fiber and phytochemicals) and the risk of NAFLD [34,35,36]. The hPDI and other healthy dietary indices such as the Mediterranean diet, Dietary Approaches to Stop Hypertension (DASH), and Healthy Eating Index-2015 (HEI-2015), share several common dietary components such as high intake of vegetables, fruits, nuts, legumes, and whole grains, and low intake of red and processed meats. These dietary indices generally showed an inverse association with odds of NAFLD [37,38,39]. In particular, the Mediterranean diet is highly regarded as the diet of choice for NAFLD in several dietary guidelines [3]. Besides weight loss, the beneficial effect of the Mediterranean Diet is partly attributable to the dietary fiber and phytochemicals with antioxidant and anti-inflammatory properties, which mostly originate from vegetables and fruits [40]. In addition, the associations between PDIs and high NFS in the current study may suggest that the hPDI was associated with low prevalence, whereas the uPDI was associated with a high prevalence, of liver fibrosis in NAFLD patients.

We found that adjustment for BMI and/or waist circumference somewhat attenuated the associations between hPDI, uPDI and the odds of NAFLD. In the subgroup analysis by BMI, however, the associations between each PDI and odds of NAFLD were similar across stratifications. Consistently, a study in northern Germany found that overall PDI and hPDI were both significantly associated with lower odds of FLD in models without BMI, and the inverse associations were largely diluted and became non-significant when additionally adjusting for BMI [13]. Similarly, upon further adjusting for BMI or waist circumference, attenuation of the associations of several dietary patterns (e.g., Mediterranean diet [41], Western diet [42], and vegetarian diet [4]) and food groups (e.g., fruits [43] and SSBs [44]) with odds/risk of FLD was found. Moreover, current evidence suggests that diet or energy intake restriction can affect the onset and/or the progression of NAFLD partly through body weight control [45]. Taken together, these findings seemingly support the hypothesis that obesity may play a mediating role in the association of diet including hPDI and uPDI with NAFLD.

The inverse association between adherence to hPDI and odds of NAFLD, as well as liver fibrosis, has biological plausibility. The hPDI recommends several healthy plant foods such as whole grains, vegetables, nuts, legumes, and coffee, which were suggested to be associated with lower odds of NAFLD and/or liver fibrosis [46,47], while the hPDI discourages several less healthy plant foods such as SSBs, which were associated with higher odds of NAFLD and liver fibrosis [44,48]. Additionally, it has been well documented that adherence to hPDI is associated with lower levels of leptin, insulin, C-reactive protein, and higher levels of adiponectin in various populations [49,50,51], while inflammation and insulin resistance play a central role in the occurrence and progression of NAFLD/liver fibrosis [52,53,54,55]. Thus, hPDI may prevent NAFLD and liver fibrosis partly through its anti-inflammatory or anti-insulin resistance property.

We found that race/ethnicity significantly modified the association between hPDI and NAFLD, with a stronger inverse association being observed in non-Hispanic whites, but not in others. A similar pattern was observed in the association between the Mediterranean diet and cognition impairment in the NHANES study [56], although the exact mechanisms for such effect modification remain unclear. Compared to non-Hispanic whites, the NAFLD risk factors such as the levels of oxidative stress and inflammation, and the prevalence of cardiovascular risk factors [52,53,57] were higher in non-Hispanic blacks and Hispanics [58,59,60]. We also compared the intake levels of food components included in the PDIs across racial and ethnic groups. By comparison, non-Hispanic whites generally consumed more nuts, plant oils, tea and coffee, and less refined grains. On the other hand, non-Hispanic whites had higher average intake levels of SSBs, potatoes, and animal fats, and lower levels of fruits and vegetables compared to those in other US populations in our study (data not shown). Therefore, the racial/ethnic differences in the hPDI-NAFLD association are likely to be partly due to the subtle difference in how a high hPDI score was comprised.

In addition, the inverse association between hPDI and NAFLD appeared stronger in males than in females in this study, although the interaction *p* value did not reach statistical significance. Similar results were obtained in the inverse association between coffee consumption and fatty liver [61]. The mean age was 49.5 years for males and 48.8 years for females in the current study. Evidence has shown that postmenopausal women have higher rates of NAFLD and severe liver fibrosis [62], which may be partially attributable to the high oxidative stress in menopausal women [63]. However, the result might be due to chance. Future research is needed to confirm this finding.

Strengths of our study include the use of a large nationally representative sample of US adults and a valid method (i.e., NAFLD via TE) with a sensitivity and specificity of both 90% for NAFLD diagnosis [16]. However, several limitations should be noted. First, the cross-sectional design does not allow the determination of causation. Second, self-reported diet and other lifestyle factors from questionnaires have measurement errors. In addition, diet assessment using 2-day dietary recalls may not accurately represent the long-term dietary intake, although the NHANES applied several methods such as the multiple-pass method and dietary sampling weight [21] to reduce dietary measurement error and improve estimates of usual intake. Third, residual dietary confounding exists. However, major potential dietary etiological factors of chronic liver diseases, such as whole grain, fruits, vegetables, nuts, potatoes, animal fat, meat, egg, dairy, fish, SSB, and coffee, were included in the construct of the PDIs. In addition, these component food groups showed a weak or a non-significant association with NAFLD in the current study (data not shown), and thus did not constitute strong confounders. Therefore, residual dietary confounding is less of a concern. Fourth, endotoxin and oxidant stress, previously shown to be altered in NAFLD, were not available in the NHANES, and thus we cannot investigate the underlying mechanism. Finally, liver biopsy in a subgroup of patients should be performed to confirm the efficacy of diet, whereas liver biopsy is also not available in the NHANES.

## 5. Conclusions

In summary, our findings suggest that greater adherence to hPDI might be associated with lower odds of NAFLD, particularly among US non-Hispanic whites. These results support the guidelines to increase healthy plant foods intake and reduce the intake of less healthy plant foods and certain animal foods in NAFLD prevention. These findings need to be validated in cohort or intervention studies. In addition, the underlying mechanisms for the racial/ethnic differences in the hPDI-NAFLD association remain to be further elucidated.

## Figures and Tables

**Table 1:** Age-adjusted characteristics of participants based on the tertiles of PDIs ^a^.

Variable	Overall PDI			hPDI			uPDI		
	Tertile 1	Tertile 2	Tertile 3	Tertile 1	Tertile 2	Tertile 3	Tertile 1	Tertile 2	Tertile 3
N	1195	1408	1297	1298	1263	1339	1406	1253	1241
PDI score, median (IQR)	48 (46–49)	53 (52–54)	58 (57–61)	48 (45–49)	53 (52–54)	59 (57–62)	48 (45–50)	54 (53–55)	60 (58–62)
Age, years	45.6 (19.4)	49.0 (18.2)	52.6 (16.9)	44.6 (18.5)	50.0 (18.6)	52.8 (17.2)	54.0 (17.2)	49.6 (18.4)	43.2 (18.0)
Female, %	50.5	51.6	54.5	42.1	52.9	60.3	52.1	53.4	50.9
BMI, kg/m^2^	29.8 (7.3)	29.7 (7.1)	28.9 (6.8)	30.4 (7.4)	29.6 (7.2)	28.4 (6.4)	28.6 (6.4)	29.6 (7.0)	30.3 (7.7)
Total energy, kcal/d	1809 (673)	1980 (723)	2133 (703)	2293 (698)	1906 (674)	1734 (633)	2013 (688)	1947 (705)	1960 (743)
Diabetes, %	17.2	17.5	15.7	15.0	17.4	17.7	16.7	15.7	18.4
Race/ethnicity, %									
Non-Hispanic white	38.7	35.3	30.8	37.9	34.6	34.0	38.6	31.5	34.3
Non-Hispanic black	27.3	23.0	18.0	29.2	24.9	13.8	14.6	24.3	29.6
Other races	34.0	41.6	51.2	33.0	40.6	52.2	46.8	44.3	36.1
Education, %									
≤12th grade	18.2	17.5	18.3	18.0	18.2	16.5	12.1	19.3	22.4
High school graduate/GED or equivalent	30.1	26.5	17.2	29.2	26.0	18.7	20.4	24.5	28.8
More than high school	51.4	56.0	64.3	52.7	55.5	64.6	67.3	56.0	48.7
Marital status, %									
Married	52.7	56.8	60.7	55.0	55.3	60.4	62.9	55.6	52.5
Widowed/divorced/separated	23.1	20.2	19.5	20.0	21.9	20.5	18.2	23.1	21.2
Never married	17.6	18.1	16.2	19.5	16.0	16.1	14.8	16.7	19.9
Ratio of family income to poverty									
<1.30	25.9	25.5	22.7	26.9	24.5	21.8	19.1	24.4	30.2
1.30–3.49	37.1	36.1	35.1	39.5	33.9	34.2	33.7	36.0	38.8
≥3.50	24.2	27.0	32.3	21.3	28.5	34.5	36.3	27.0	19.7
Physical activity, METS–h/week									
<8.3	34.7	35.0	34.3	34.7	36.8	32.4	28.3	36.9	39.3
8.3–16.7	9.6	7.9	11.3	8.0	8.7	11.8	11.2	8.8	8.2
>16.7	54.6	56.1	54.0	56.4	53.5	55.3	60.1	53.2	51.7
Smoking, %									
Never smokers	56.9	60.2	66.1	55.9	60.8	66.1	64.7	61.4	57.8
Former smokers	23.5	23.3	23.5	23.9	23.7	23.3	25.0	23.0	21.9
Current smokers	19.6	16.5	10.4	20.2	15.5	10.6	10.3	15.7	20.2
Alcohol drinking, %									
Never drinkers	11.6	10.0	11.2	9.5	10.3	12.5	11.3	10.3	11.2
Former drinkers	19.6	19.4	19.4	21.5	19.2	17.9	15.4	20.8	24.1
Current drinkers	65.9	68.7	66.1	67.1	68.0	66.3	70.4	66.6	62.1

GED, general educational development; hPDI, healthful plant-based diet index; IQR, interquartile range; METS, metabolic equivalent tasks; PDI, plant-based diets index; uPDI, unhealthful plant-based diet index. ^a^ Continuous variables were presented as means (standard deviation) or median (IQR). Variables were standardized based on the study population’s age distribution except for age and PDIs scores. The percentage within some categories may not sum to 100 due to rounding or missing values.

**Table 2:** Associations of the plant-based diets with NAFLD.

	OR (95% CI)				*p*_trend_^d^
	Tertile 1	Tertile 2	Tertile 3	Per 10–Point Increase	
Overall PDI					
Model 1 ^a^	Reference	1.02 (0.77, 1.35)	0.99 (0.75, 1.30)	0.96 (0.78, 1.18)	0.673
Model 2 ^b^	Reference	0.94 (0.70, 1.24)	0.88 (0.67, 1.16)	0.86 (0.69, 1.06)	0.147
Model 3 ^c^	Reference	0.92 (0.66, 1.28)	1.03 (0.76, 1.38)	1.06 (0.86, 1.30)	0.609
hPDI					
Model 1 ^a^	Reference	0.77 (0.62, 0.97)	0.53 (0.39, 0.72)	0.62 (0.49, 0.78)	**<0.001**
Model 2 ^b^	Reference	0.74 (0.57, 0.97)	0.50 (0.35, 0.72)	0.59 (0.46, 0.77)	**<0.001**
Model 3 ^c^	Reference	0.78 (0.58, 1.06)	0.64 (0.46, 0.87)	0.74 (0.59, 0.92)	**0.006**
uPDI					
Model 1 ^a^	Reference	1.20 (0.93, 1.54)	1.32 (0.89, 1.95)	1.29 (1.02, 1.62)	0.034
Model 2 ^b^	Reference	1.25 (0.98, 1.59)	1.37 (0.93, 2.02)	1.37 (1.08, 1.74)	**0.009**
Model 3 ^c^	Reference	1.18 (0.93, 1.51)	1.14 (0.79, 1.66)	1.16 (0.94, 1.45)	0.173

CI, confidence interval; hPDI, healthful plant-based diet index; NAFLD, nonalcoholic fatty liver disease; OR, odds ratio; PDI, plant-based diets index; uPDI, unhealthful plant-based diet index. ^a^ Model 1 adjusted for age only. ^b^ Model 2 was further adjusted for sex, ratio of family income to poverty, race/ethnicity, total energy intake, marital status, education, smoking, alcohol drinking, diabetes, and physical activity. ^c^ Model 3 was further adjusted for body mass index. ^d^ *p* values in bold font meet a Bonferroni-corrected significance level.

## Data Availability

The data used in this study are openly available in: https://www.cdc.gov/nchs/nhanes/index.htm (accessed on 10 May 2021).

## Supplementary Materials

The following supporting information can be downloaded at: www.mdpi.com/article/10.3390/nu14194099/s1. Figure S1: Flow chart of selection of participants in the analysis. Table S1: Food components in three plant-based dietary indices. Table S2: Details of categorizations of the covariates. Table S3: Dietary intake of people according to adherence to the plant-based diets in NHANES (2017–2018). Table S4: Sensitivity analyses on the association between plant-based diets and odds of NAFLD using different cut-off value (CAP ≥ 288 dB/m). Table S5: Sensitivity analyses on the association between plant-based diets and odds of NAFLD defined by US fatty liver index (US FLI ≥ 30). Table S6: Sensitivity analyses on the association between plant-based diets and odds of NAFLD in people not having diabetes or prediabetes. Table S7: Sensitivity analyses on the association between plant-based diets and odds of NAFLD by treating the numerical covariates as continuous variables. Table S8: Sensitivity analyses on the association between plant-based diets and odds of NAFLD by additionally adjusting for waist circumference. Table S9: Sensitivity analyses on the association between plant-based diets and high NAFLD fibrosis score (NFS > 0.676) in NAFLD patients. Table S10: Sensitivity analyses on the association between plant-based diets and high fibrosis-4 index (FIB-4 > 3.25) in NAFLD patients.
