# Supplementary material for: A Healthful Plant-Based Diet Is Associated with Lower Odds of Nonalcoholic Fatty Liver Disease
Source: Nutrients. 2022 Oct 2;14(19):4099. doi: 10.3390/nu14194099 (PMC9572274; doi:10.3390/nu14194099)
Supplement: Supplementary file 1 [file nutrients-14-04099-s001.zip › nutrients-1887808-supplementary.pdf]

Figure S1. Flow chart of selection of participants in the analysis

Table S1. Food components in three plant-based dietary indices

Table S2. Details of categorizations of adjusted covariates

Table S3. Dietary intake of people according to adherence to the plant-based diets in NHANES (2017-2018)

Table S4. Sensitivity analyses on the association between plant-based diets and odds of NAFLD using different cut-off value (CAP ≥ 288 dB/m)

Table S5. Sensitivity analyses on the association between plant-based diets and odds of NAFLD defined by US fatty liver index (US FLI ≥ 30)

Table S6. Sensitivity analyses on the association between plant-based diets and odds of NAFLD in people not having diabetes or prediabetes

Table S7. Sensitivity analyses on the association between plant-based diets and odds of NAFLD by treating the numerical covariates as continuous variables

Table S8. Sensitivity analyses on the association between plant-based diets and odds of NAFLD by additionally adjusting for waist circumference

Table S9. Sensitivity analyses on the association between plant-based diets and high NAFLD fibrosis score (NFS > 0.676) in NAFLD patients

Table S10. Sensitivity analyses on the association between plant-based diets and high fibrosis-4 index (FIB-4 > 3.25) in NAFLD patients

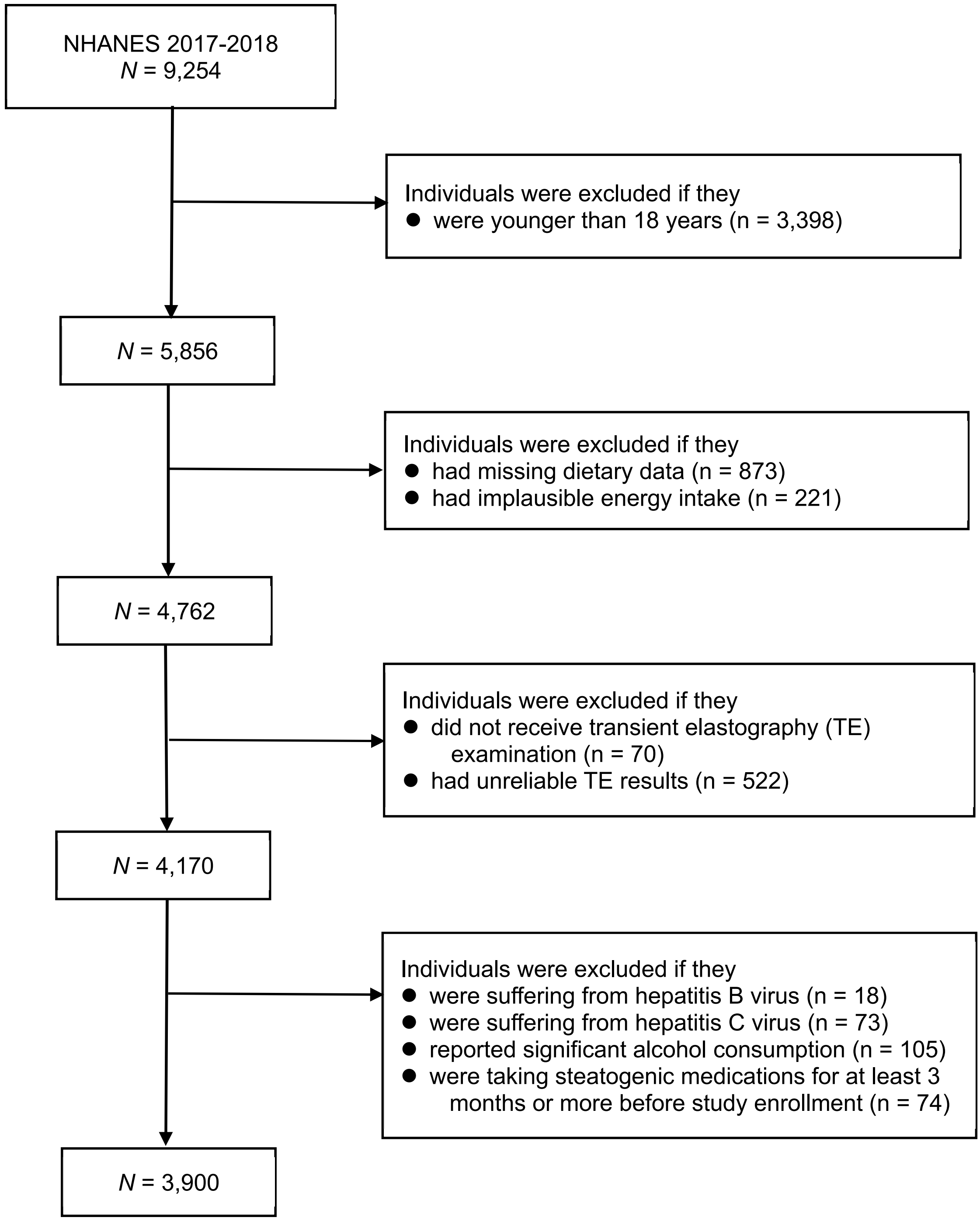

Figure S1. Flow chart of selection of participants in the analysis. NHANES, National Health and Nutrition Examination Survey; TE, transient elastography

Table S1. Food components in three plant-based dietary indices

Healthy plant foods	Food components
Whole grains	Whole grain breakfast cereal, other cooked breakfast cereal, cooked oatmeal, dark bread, brown rice, other grains, bran, wheat germ, popcorn
Fruits	Raisins or grapes, prunes, bananas, cantaloupe, watermelon, fresh apples or pears, oranges, grapefruit, strawberries, blueberries, peaches or apricots or plums
Vegetables	Tomatoes, tomato juice, tomato sauce, broccoli, cabbage, cauliflower, brussels sprouts, carrots, mixed vegetables, yellow or winter squash, eggplant or zucchini, yams or sweet potatoes, spinach cooked, spinach raw, kale or mustard orchard greens, iceberg or head lettuce, romaine or leaf lettuce, celery, mushrooms, beets, alfalfa sprouts, garlic, corn
Nuts	Nuts, peanut butter
Legumes	String beans, tofu or soybeans, beans or lentils, peas, or lima beans
Vegetable oils	Oil-based salad dressing, vegetable oil used for cooking
Tea and coffee	Tea, coffee, decaffeinated coffee
Less healthy plant foods	
Fruit juices	Apple cider (nonalcoholic) or juice, orange juice, grapefruit juice, other fruit juice
Refined grains	Refined grain breakfast cereal, white bread, English muffins or bagels or rolls, muffins or biscuits, white rice, pancakes or waffles, crackers, pasta
Potatoes	Baked, boiled, mashed, scalloped, and fried potatoes, potato chips, etc
Sugar-sweetened beverages	Colas with caffeine and sugar, colas without caffeine but with sugar, other carbonated beverages with sugar, noncarbonated fruit drinks with sugar
Sweets and desserts	Chocolates, candy bars, candy without chocolate, cookies (home-baked and ready-made), brownies, doughnuts, cake (home-baked and ready-made), sweet roll (home-baked and ready-made), pie (home-baked and ready-made), jams or jellies or preserves or syrup or honey
Animal foods	
Animal fat	Butter added to food, butter or lard used for cooking
Dairy	Skim low fat milk, whole milk, cream, sour cream, sherbet, ice cream, yogurt, cottage or ricotta cheese, cream cheese, other cheese
Egg	Eggs
Fish or seafood	Canned tuna, dark meat fish, other fish, shrimp or lobster or scallops
Meat	Chicken or turkey with skin, chicken or turkey without skin, bacon, hot dogs, processed meats, liver, hamburger, beef or pork or lamb mixed dish, beef or pork or lamb main dish
Miscellaneous animal-based foods	Pizza, chowder or cream soup, mayonnaise, or other creamy salad dressing

Table S2. Details of categorizations of the covariates

Variable name	Variable categorizations
Age	18-39, 40-59, ≥ 60 years
Sex	Male, female
Ratio of family income to poverty	< 1.30, 1.30-3.49, ≥ 3.50
Race/ethnicity	Non-Hispanic white, non-Hispanic black, other races
Total energy intake	Tertile 1, Tertile 2, Tertile 3
Marital status	Married, widowed/divorced/separated, never married
Education	12th grade, high school graduate/GED or equivalent, more than high school
Smoking	Never smokers, former smokers, current smokers
Alcohol drinking	Never drinkers, former drinkers, current drinkers
Diabetes	No, yes
Physical activity	< 8.3, 8.3-16.7, > 16.7 METS-h/week
Body mass index	< 18.5, 18.5-24.9, 25.0-29.9, ≥ 30.0 kg/m^2

GED, general educational development; METS, metabolic equivalent tasks

Table S3. Dietary intake of people according to adherence to the plant-based diets in NHANES (2017-2018)[a]

	Overall PDI			hPDI			uPDI		
Food groups	Tertile 1	Tertile 2	Tertile 3	Tertile 1	Tertile 2	Tertile 3	Tertile 1	Tertile 2	Tertile 3
Healthy plant foods									
Whole grains	17.9 (61.8)	28.2 (114.8)	48.1 (97.7)	17.8 (67.5)	23.0 (56.4)	53.3 (137.2)	52.7 (129.7)	27.4 (78.6)	12.2 (54.0)
Fruits	59.1 (113.2)	87.6 (127.3)	143.6 (150.6)	54.3 (103.8)	85.2 (117.1)	151.0 (160.4)	149.6 (156.7)	86.8 (127.8)	49.2 (91.4)
Vegetables	76.6 (114.4)	108.7 (143.3)	174.7 (178.4)	67.2 (104.9)	105.2 (128.8)	187.5 (187.1)	181.4 (168.9)	113.4 (147.6)	59.8 (109.4)
Nuts	4.1 (20.8)	11.9 (39.9)	21.9 (51.6)	4.8 (19.7)	9.4 (30.9)	24.0 (57.5)	22.4 (53.3)	10.7 (37.0)	4.2 (19.4)
Legumes	7.2 (44.8)	17.4 (48.8)	54.8 (103.1)	10.2 (43.4)	20.1 (58.7)	49.0 (99.5)	42.4 (90.8)	23.8 (69.9)	11.8 (47.5)
Vegetable oils	0.1 (1.3)	0.1 (0.9)	0.4 (2.3)	0.0 (0.5)	0.1 (1.1)	0.4 (2.4)	0.4 (2.4)	0.1 (1.1)	0.0 (0.5)
Tea and coffee	318.7 (636.5)	430.2 (520.3)	517.9 (467.4)	305.1 (479.5)	435.8 (569.6)	531.6 (567.4)	562.9 (568.2)	415.2 (513.9)	279.3 (519.8)
Less healthy plant foods									
Fruit juices	25.0 (91.8)	47.5 (115.4)	72.3 (147.3)	73.3 (145.6)	47.9 (118.5)	26.1 (93.3)	33.9 (97.7)	52.5 (126.6)	62.2 (140.0)
Refined grains	128.7 (227.5)	209.4 (306.6)	252.4 (318.9)	238.4 (311.2)	193.7 (283.6)	165.7 (280.1)	136.8 (215.1)	203.0 (302.4)	265.3 (342.2)
Potatoes	33.1 (63.0)	45.7 (71.5)	49.9 (74.0)	65.0 (76.1)	41.7 (70.8)	23.6 (56.3)	32.2 (66.5)	43.6 (69.7)	55.4 (72.8)
Sugar-sweetened beverages	242.9 (420.6)	287.1 (414.0)	246.6 (342.1)	440.0 (485.4)	236.4 (352.8)	108.0 (227.4)	110.1 (219.0)	235.0 (345.0)	455.3 (499.5)
Sweets and desserts	30.5 (48.7)	43.5 (55.2)	58.3 (58.8)	59.8 (60.7)	41.7 (54.6)	32.0 (47.5)	36.1 (48.9)	42.9 (53.9)	55.4 (62.5)
Animal foods									
Animal fat	1.3 (3.9)	1.1 (3.3)	1.1 (3.7)	1.5 (4.1)	1.1 (3.4)	0.9 (3.3)	1.7 (4.2)	1.2 (3.8)	0.5 (2.3)
Dairy	185.3 (202.4)	155.4 (181.5)	142.5 (183.9)	185.4 (202.6)	155.7 (181.7)	140.1 (181.4)	207.7 (193.2)	163.6 (195.5)	103.2 (162.5)
Egg	39.4 (54.6)	29.5 (49.4)	20.2 (37.7)	38.5 (53.1)	26.2 (45.8)	23.7 (44.0)	41.3 (52.4)	27.3 (46.5)	18.2 (41.4)
Fish or seafood	18.9 (59.1)	16.7 (48.4)	17.7 (55.7)	17.4 (55.2)	18.3 (55.7)	17.5 (52.1)	23.5 (54.7)	17.9 (61.8)	11.0 (44.0)
Meats	95.3 (85.5)	84.1 (88.8)	62.1 (77.8)	104.0 (91.8)	78.7 (84.7)	58.5 (72.4)	89.9 (83.4)	79.2 (86.3)	70.2 (85.3)
Miscellaneous animal-based foods	186.4 (184.3)	142.4 (169.5)	91.9 (133.9)	184.5 (187.6)	142.2 (164.6)	92.1 (134.8)	138.0 (152.1)	139.4 (169.4)	140.0 (182.7)

hPDI, Healthful plant-based diet index; NHANES, National Health and Nutrition Examination Survey; PDI, Plant-based diet index; SD, Standard deviation; uPDI, Unhealthful plant-based diet index.
[a] Variables were expressed as mean (SD) if they were normally distributed

Table S4. Sensitivity analyses on the association between plant-based diets and odds of NAFLD using different cut-off value (CAP ≥ 288 dB/m)

	OR (95% CI)				P_{trend} [d]
	Tertile 1	Tertile 2	Tertile 3	Per 10-point increase	
**Overall PDI**					
Model 1[a]	Reference	1.05 (0.78, 1.43)	0.92 (0.67, 1.26)	0.93 (0.74, 1.17)	0.517
Model 2[b]	Reference	0.98 (0.72, 1.33)	0.84 (0.60, 1.17)	0.85 (0.66, 1.08)	0.175
Model 3[c]	Reference	0.98 (0.72, 1.35)	0.97 (0.67, 1.38)	1.04 (0.82, 1.31)	0.773
**hPDI**					
Model 1[a]	Reference	0.72 (0.58, 0.91)	0.48 (0.34, 0.66)	0.59 (0.47, 0.75)	< 0.001
Model 2[b]	Reference	0.69 (0.51, 0.93)	0.46 (0.31, 0.68)	0.58 (0.43, 0.77)	< 0.001
Model 3[c]	Reference	0.72 (0.51, 1.02)	0.57 (0.39, 0.84)	0.72 (0.55, 0.94)	0.014
**uPDI**					
Model 1[a]	Reference	1.19 (0.92, 1.55)	1.26 (0.83, 1.91)	1.26 (0.99, 1.61)	0.065
Model 2[b]	Reference	1.24 (0.95, 1.63)	1.27 (0.82, 1.97)	1.31 (1.01, 1.71)	0.046
Model 3[c]	Reference	1.17 (0.91, 1.50)	1.05 (0.69, 1.59)	1.11 (0.86, 1.42)	0.429

CAP, Controlled attenuation parameter; CI, Confidence interval; hPDI, Healthful plant-based diet index; NAFLD, Nonalcoholic fatty liver disease; OR, Odds ratio; PDI, Plant-based diets index; uPDI, Unhealthful plant-based diet index

[a] Model 1 was adjusted for age (18-39, 40-59, and ≥ 60 years)

[b] Model 2 was further adjusted for sex (male, female), total energy intake (kcal/day, tertile), race/ethnicity (non-Hispanic white, non-Hispanic black, or other races), education (≤ 12th grade, high school graduate/GED or equivalent, or more than high school), marital status (married, widowed/divorced/separated, or never married), ratio of family income to poverty (< 1.30, 1.30-3.49, or ≥ 3.50), physical activity (< 8.3, 8.3-16.7, or > 16.7 METS-h/week), smoking (never smokers, former smokers, or current smokers), alcohol drinking (never drinkers, former drinkers, or current drinkers), and diabetes (no, yes)

[c] Model 3 was further adjusted for body mass index (< 18.5, 18.5-24.9, 25.0-29.9, and ≥ 30.0 kg/m^2)

[d] Linear trend test was conducted by treating each PDI as a continuous variable in the models

Table S5. Sensitivity analyses on the association between plant-based diets and odds of NAFLD defined by US fatty liver index (US FLI ≥ 30)

	OR (95% CI)				P_{trend} [d]
	Tertile 1	Tertile 2	Tertile 3	Per 10-point increase	
**Overall PDI**					
Model 1[a]	Reference	1.11 (0.76, 1.62)	0.96 (0.62, 1.48)	0.91 (0.63, 1.31)	0.601
Model 2[b]	Reference	1.03 (0.67, 1.58)	0.86 (0.51, 1.45)	0.79 (0.50, 1.25)	0.316
Model 3[c]	Reference	1.09 (0.67, 1.78)	0.94 (0.52, 1.70)	0.90 (0.54, 1.51)	0.701
**hPDI**					
Model 1[a]	Reference	0.78 (0.52, 1.19)	0.46 (0.30, 0.71)	0.56 (0.41, 0.77)	< 0.001
Model 2[b]	Reference	0.70 (0.44, 1.10)	0.39 (0.25, 0.62)	0.48 (0.33, 0.69)	< 0.001
Model 3[c]	Reference	0.73 (0.40, 1.34)	0.50 (0.33, 0.78)	0.64 (0.44, 0.92)	0.017
**uPDI**					
Model 1[a]	Reference	1.46 (0.94, 2.25)	1.51 (0.99, 2.30)	1.42 (1.11, 1.81)	0.005
Model 2[b]	Reference	1.53 (1.01, 2.30)	1.78 (1.07, 2.98)	1.73 (1.29, 2.34)	< 0.001
Model 3[c]	Reference	1.10 (0.75, 1.61)	1.41 (0.79, 2.52)	1.41 (1.03, 1.91)	0.026

CI, Confidence interval; FLI, Fatty liver index; hPDI, Healthful plant-based diet index; NAFLD, Nonalcoholic fatty liver disease; OR, Odds ratio; PDI, Plant-based diets index; uPDI, Unhealthful plant-based diet index

[a] Model 1 was adjusted for age (18-39, 40-59, and ≥ 60 years)

[b] Model 2 was further adjusted for sex (male, female), total energy intake (kcal/day, tertile), race/ethnicity (non-Hispanic white, non-Hispanic black, or other races), education (≤ 12th grade, high school graduate/GED or equivalent, or more than high school), marital status (married, widowed/divorced/separated, or never married), ratio of family income to poverty (< 1.30, 1.30-3.49, or ≥ 3.50), physical activity (< 8.3, 8.3-16.7, or > 16.7 METS-h/week), smoking (never smokers, former smokers, or current smokers), alcohol drinking (never drinkers, former drinkers, or current drinkers), and diabetes (no, yes)

[c] Model 3 was further adjusted for body mass index (< 18.5, 18.5-24.9, 25.0-29.9, and ≥ 30.0 kg/m^2)

[d] Linear trend test was conducted by treating each PDI as a continuous variable in the models

Table S6. Sensitivity analyses on the association between plant-based diets and odds of NAFLD in people not having diabetes or prediabetes

	OR (95% CI)				P_{trend} [d]
	Tertile 1	Tertile 2	Tertile 3	Per 10-point increase	
**Overall PDI**					
Model 1[a]	Reference	1.02 (0.76, 1.37)	0.96 (0.73, 1.26)	0.95 (0.75, 1.20)	0.680
Model 2[b]	Reference	0.92 (0.67, 1.27)	0.84 (0.64, 1.11)	0.85 (0.68, 1.06)	0.145
Model 3[c]	Reference	0.87 (0.61, 1.25)	1.01 (0.76, 1.33)	1.07 (0.87, 1.32)	0.517
**hPDI**					
Model 1[a]	Reference	0.79 (0.62, 1.01)	0.51 (0.37, 0.71)	0.60 (0.47, 0.76)	< 0.001
Model 2[b]	Reference	0.81 (0.63, 1.03)	0.50 (0.35, 0.72)	0.59 (0.45, 0.77)	< 0.001
Model 3[c]	Reference	0.91 (0.68, 1.21)	0.63 (0.45, 0.87)	0.72 (0.57, 0.91)	0.007
**uPDI**					
Model 1[a]	Reference	1.28 (0.99, 1.66)	1.44 (0.93, 2.22)	1.38 (1.06, 1.81)	0.017
Model 2[b]	Reference	1.32 (1.00, 1.73)	1.48 (0.97, 2.27)	1.46 (1.12, 1.90)	0.005
Model 3[c]	Reference	1.27 (0.95, 1.69)	1.24 (0.80, 1.92)	1.26 (0.97, 1.64)	0.080

CI, Confidence interval; hPDI, Healthful plant-based diet index; NAFLD, Nonalcoholic fatty liver disease; OR, Odds ratio; PDI, Plant-based diets index; uPDI, Unhealthful plant-based diet index

[a] Model 1 was adjusted for age (18-39, 40-59, and ≥ 60 years)

[b] Model 2 was further adjusted for sex (male, female), total energy intake (kcal/day, tertile), race/ethnicity (non-Hispanic white, non-Hispanic black, or other races), education (≤ 12th grade, high school graduate/GED or equivalent, or more than high school), marital status (married, widowed/divorced/separated, or never married), ratio of family income to poverty (< 1.30, 1.30-3.49, or ≥ 3.50), physical activity (< 8.3, 8.3-16.7, or > 16.7 METS-h/week), smoking (never smokers, former smokers, or current smokers), and alcohol drinking (never drinkers, former drinkers, or current drinkers)

[c] Model 3 was further adjusted for body mass index (< 18.5, 18.5-24.9, 25.0-29.9, and ≥ 30.0 kg/m^2)

[d] Linear trend test was conducted by treating each PDI as a continuous variable in the models

Table S7. Sensitivity analyses on the association between plant-based diets and odds of NAFLD by treating the numerical covariates as continuous variables

	OR (95% CI)				P_{trend} [d]
	Tertile 1	Tertile 2	Tertile 3	Per 10-point increase	
**Overall PDI**					
Model 1[a]	Reference	1.02 (0.77, 1.36)	0.95 (0.73, 1.25)	0.94 (0.77, 1.15)	0.520
Model 2[b]	Reference	0.92 (0.68, 1.24)	0.87 (0.65, 1.18)	0.84 (0.67, 1.07)	0.155
Model 3[c]	Reference	0.93 (0.65, 1.32)	1.03 (0.78, 1.36)	1.04 (0.86, 1.27)	0.672
**hPDI**					
Model 1[a]	Reference	0.76 (0.61, 0.93)	0.52 (0.38, 0.70)	0.60 (0.48, 0.76)	< 0.001
Model 2[b]	Reference	0.68 (0.51, 0.91)	0.46 (0.30, 0.70)	0.55 (0.40, 0.77)	< 0.001
Model 3[c]	Reference	0.74 (0.53, 1.01)	0.60 (0.40, 0.90)	0.70 (0.52, 0.95)	0.020
**uPDI**					
Model 1[a]	Reference	1.22 (0.95, 1.56)	1.40 (0.95, 2.06)	1.34 (1.06, 1.70)	0.014
Model 2[b]	Reference	1.37 (1.06, 1.78)	1.57 (1.05, 2.33)	1.54 (1.20, 1.97)	0.001
Model 3[c]	Reference	1.23 (0.96, 1.58)	1.23 (0.86, 1.75)	1.28 (1.03, 1.60)	0.027

CI, Confidence interval; hPDI, Healthful plant-based diet index; NAFLD, Nonalcoholic fatty liver disease; OR, Odds ratio; PDI, Plant-based diets index; uPDI, Unhealthful plant-based diet index

[a] Model 1 was adjusted for age (continuous, years)

[b] Model 2 was further adjusted for sex (male, female), total energy intake (continuous, kcal/day), race/ethnicity (non-Hispanic white, non-Hispanic black, or other races), education (≤ 12th grade, high school graduate/GED or equivalent, or more than high school), marital status (married, widowed/divorced/separated, or never married), ratio of family income to poverty (continuous), physical activity (continuous, METS-h/week), smoking (never smokers, former smokers, or current smokers), and alcohol drinking (never drinkers, former drinkers, or current drinkers)

[c] Model 3 was further adjusted for body mass index (continuous, kg/m^2)

[d] Linear trend test was conducted by treating each PDI as a continuous variable in the models

Table S8. Sensitivity analyses on the association between plant-based diets and odds of NAFLD by additionally adjusting for waist circumference

	OR (95% CI) [a]				P_{trend} [b]
	Tertile 1	Tertile 2	Tertile 3	Per 10-point increase	
Overall PDI	Reference	0.94 (0.67, 1.32)	1.04 (0.76, 1.42)	1.08 (0.87, 1.34)	0.488
hPDI	Reference	0.81 (0.59, 1.13)	0.69 (0.48, 0.97)	0.76 (0.60, 0.97)	0.028
uPDI	Reference	1.13 (0.89, 1.44)	1.06 (0.73, 1.56)	1.12 (0.88, 1.41)	0.350

CI, Confidence interval; hPDI, Healthful plant-based diet index; NAFLD, Nonalcoholic fatty liver disease; OR, Odds ratio; PDI, Plant-based diets index; uPDI, Unhealthful plant-based diet index

[a] Models were adjusted for age (18-39, 40-59, and ≥ 60 years), sex (male, female), total energy intake (kcal/day, tertile), race/ethnicity (non-Hispanic white, non-Hispanic black, or other races), education (≤ 12th grade, high school graduate/GED or equivalent, or more than high school), marital status (married, widowed/divorced/separated, or never married), ratio of family income to poverty (< 1.30, 1.30-3.49, or ≥ 3.50), physical activity (< 8.3, 8.3-16.7, or > 16.7 METS-h/week), smoking (never smokers, former smokers, or current smokers), and alcohol drinking (never drinkers, former drinkers, or current drinkers), body mass index (< 18.5, 18.5-24.9, 25.0-29.9, and ≥ 30.0 kg/m^2), and waist circumference (continuous, cm).

[b] Linear trend test was conducted by treating each PDI as a continuous variable in the models

Table S9. Sensitivity analyses on the association between plant-based diets and high NAFLD fibrosis score (NFS > 0.676) in NAFLD patients

	OR (95% CI)				P_{trend} [d]
	Tertile 1	Tertile 2	Tertile 3	Per 10-point increase	
**Overall PDI**					
Model 1[a]	Reference	1.19 (0.63, 2.23)	0.94 (0.56, 1.58)	0.78 (0.56, 1.08)	0.132
Model 2[b]	Reference	1.29 (0.63, 2.64)	1.16 (0.59, 2.27)	0.95 (0.68, 1.32)	0.748
Model 3[c]	Reference	1.35 (0.63, 2.85)	1.27 (0.64, 2.49)	1.05 (0.75, 1.48)	0.770
**hPDI**					
Model 1[a]	Reference	0.93 (0.58, 1.50)	0.46 (0.33, 0.62)	0.53 (0.37, 0.77)	0.001
Model 2[b]	Reference	1.00 (0.61, 1.62)	0.49 (0.34, 0.73)	0.57 (0.38, 0.84)	0.004
Model 3[c]	Reference	0.99 (0.59, 1.67)	0.58 (0.37, 0.89)	0.64 (0.43, 0.96)	0.030
**uPDI**					
Model 1[a]	Reference	1.34 (0.79, 2.27)	1.84 (0.98, 3.46)	1.79 (1.21, 2.66)	0.004
Model 2[b]	Reference	1.69 (0.75, 3.80)	2.02 (1.06, 3.83)	1.98 (1.32, 2.98)	0.001
Model 3[c]	Reference	1.66 (0.69, 4.02)	1.96 (0.97, 3.96)	1.86 (1.20, 2.88)	0.005

CAP, Controlled attenuation parameter; CI, Confidence interval; hPDI, Healthful plant-based diet index; NAFLD, Nonalcoholic fatty liver disease; NFS, NAFLD fibrosis score; OR, Odds ratio; PDI, Plant-based diets index; uPDI, Unhealthful plant-based diet index

[a] Model 1 was adjusted for age (18-39, 40-59, and ≥ 60 years)

[b] Model 2 was further adjusted for sex (male, female), total energy intake (kcal/day, tertile), race/ethnicity (non-Hispanic white, non-Hispanic black, or other races), education (≤ 12th grade, high school graduate/GED or equivalent, or more than high school), marital status (married, widowed/divorced/separated, or never married), ratio of family income to poverty (< 1.30, 1.30-3.49, or ≥ 3.50), physical activity (< 8.3, 8.3-16.7, or > 16.7 METS-h/week), smoking (never smokers, former smokers, or current smokers), alcohol drinking (never drinkers, former drinkers, or current drinkers), and diabetes (no, yes)

[c] Model 3 was further adjusted for body mass index (< 18.5, 18.5-24.9, 25.0-29.9, and ≥ 30.0 kg/m^2)

[d] Linear trend test was conducted by treating each PDI as a continuous variable in the models

Table S10. Sensitivity analyses on the association between plant-based diets and high fibrosis-4 index (FIB-4 > 3.25) in NAFLD patients

	OR (95% CI)				P_{trend} [d]
	Tertile 1	Tertile 2	Tertile 3	Per 10-point increase	
**Overall PDI**					
Model 1[a]	Reference	0.34 (0.09, 1.26)	0.17 (0.05, 0.61)	0.21 (0.08, 0.55)	0.001
Model 2[b]	Reference	0.28 (0.07, 1.10)	0.20 (0.07, 0.56)	0.24 (0.10, 0.56)	0.001
Model 3[c]	Reference	0.30 (0.08, 1.15)	0.20 (0.07, 0.56)	0.21 (0.09, 0.51)	0.001
**hPDI**					
Model 1[a]	Reference	0.34 (0.10, 1.15)	0.51 (0.14, 1.78)	0.74 (0.25, 2.21)	0.586
Model 2[b]	Reference	0.23 (0.08, 0.70)	0.37 (0.12, 1.17)	0.61 (0.22, 1.70)	0.346
Model 3[c]	Reference	0.26 (0.09, 0.79)	0.32 (0.10, 1.03)	0.53 (0.25, 1.16)	0.114
**uPDI**					
Model 1[a]	Reference	2.85 (0.76, 10.68)	1.61 (0.28, 9.32)	1.28 (0.44, 3.69)	0.653
Model 2[b]	Reference	3.31 (1.04, 10.58)	1.70 (0.30, 9.67)	1.24 (0.43, 3.58)	0.694
Model 3[c]	Reference	3.37 (1.30, 8.78)	1.97 (0.38, 10.17)	1.38 (0.52, 3.66)	0.520

CI, Confidence interval; FIB-4, Fibrosis-4 index; hPDI, Healthful plant-based diet index; NAFLD, Nonalcoholic fatty liver disease; OR, Odds ratio; PDI, Plant-based diets index; uPDI, Unhealthful plant-based diet index

[a] Model 1 was adjusted for age (18-39, 40-59, and ≥ 60 years)

[b] Model 2 was further adjusted for sex (male, female), total energy intake (kcal/day, tertile), race/ethnicity (non-Hispanic white, non-Hispanic black, or other races), education (≤ 12th grade, high school graduate/GED or equivalent, or more than high school), marital status (married, widowed/divorced/separated, or never married), ratio of family income to poverty (< 1.30, 1.30-3.49, or ≥ 3.50), physical activity (< 8.3, 8.3-16.7, or > 16.7 METS-h/week), smoking (never smokers, former smokers, or current smokers), alcohol drinking (never drinkers, former drinkers, or current drinkers), and diabetes (no, yes)

[c] Model 3 was further adjusted for body mass index (< 18.5, 18.5-24.9, 25.0-29.9, and ≥ 30.0 kg/m^2)

[d] Linear trend test was conducted by treating each PDI as a continuous variable in the models
